# Optofluidic Sensor Based on Polymer Optical Microresonators for the Specific, Sensitive and Fast Detection of Chemical and Biochemical Species

**DOI:** 10.3390/s23177373

**Published:** 2023-08-24

**Authors:** Nolwenn-Amandine Keriel, Camille Delezoide, David Chauvin, Hafsa Korri-Youssoufi, Ngoc Diep Lai, Isabelle Ledoux-Rak, Chi-Thanh Nguyen

**Affiliations:** 1 Laboratoire Lumière, Matière et Interfaces (LuMIn), Ecole Normale Superieure Paris Saclay, Centre National de la Recherche Scientifique, Unité Mixte de Recherche 9024, CentraleSupelec, Institut d’Alembert, Université Paris Saclay, 4 Avenue des Sciences, 91190 Gif-sur-Yvette, France; k-amandina@hotmail.com (N.-A.K.); ngoc-diep.lai@ens-paris-saclay.fr (N.D.L.); chi-thanh.nguyen@ens-cachan.fr (C.-T.N.); 2 Institut de Chimie Moléculaire et des Matériaux d’Orsay (ICMMO), Centre National de la Recherche Scientifique, Unité Mixte de Recherche 8182, Université Paris Saclay, 17 Avenue des Sciences, 91400 Orsay, France; hafsa.korri-youssoufi@universite-paris-saclay.fr

**Keywords:** microresonator, DNA detection, optofluidics, polymer-based waveguides

## Abstract

The accurate, rapid, and specific detection of DNA strands in solution is becoming increasingly important, especially in biomedical applications such as the trace detection of COVID-19 or cancer diagnosis. In this work we present the design, elaboration and characterization of an optofluidic sensor based on a polymer-based microresonator which shows a quick response time, a low detection limit and good sensitivity. The device is composed of a micro-racetrack waveguide vertically coupled to a bus waveguide and embedded within a microfluidic circuit. The spectral response of the microresonator, in air or immersed in deionised water, shows quality factors up to 72,900 and contrasts up to 0.9. The concentration of DNA strands in water is related to the spectral shift of the microresonator transmission function, as measured at the inflection points of resonance peaks in order to optimize the signal-over-noise ratio. After functionalization by a DNA probe strand on the surface of the microresonator, a specific and real time measurement of the complementary DNA strands in the solution is realized. Additionally, we have inferred the dissociation constant value of the binding equilibrium of the two complementary DNA strands and evidenced a sensitivity of 16.0 pm/µM and a detection limit of 121 nM.

## 1. Introduction

### 1.1. Presentation and State-of-the Art

The emergence of the COVID-19 pandemic on one hand and the constant need for early and non-invasive cancer diagnosis on the other hand has made the development of new tools for biomolecular detection an urgent priority. The specific detection of nucleic acids is mainly based on PCR technology [1], identification of antibodies [2], electrochemistry [3] or optical detection methods [4].

Label-based techniques, mainly using fluorescent markers, have been used in commercial tools such as PCR and ELISA tests for a long time. However, they display slow response and require expensive equipment [5]. Moreover, labelling may alter the physico-chemical properties of the marked molecules and perturb their recognition process. For these reasons, different label-free detection methods have been proposed, some of them currently being developed for commercial use. Various physical phenomena may be involved in such recognition processes: mass variation using quartz microbalance [6], electrochemical sensors [7,8], plasmon resonance-based sensors [9,10], photonic crystals [11], optical fibers [12] and microresonators [13,14].

Most of label-free optical sensors are based on refractive index modifications induced by the detection process at the interface separating two dielectric media, one of them (medium of interest) containing the molecule (target) to be detected. In optical devices involving total internal reflection or waveguiding, their response depends on the interaction between this evanescent wave and the target molecules within the medium of interest. The presence and concentration of target moieties modifies the refractive index of the medium of interest and, hence, the device optical properties. As compared to PCR, DNA sensors based on optofluidic devices provide real-time measurements without amplification and do so using handheld devices suitable for point-of-care diagnosis.

A first class of these refractive index-dependent phenomena is related to interferences, which have been used in two kinds of waveguide interferometric sensors: Mach–Zehnder and Young interferometers [15]. These devices display two arms, one of them being buried within a protective layer, and the second being exposed to the target species to be detected. Another class is focused on optical fiber sensors, involving, in general, Bragg gratings which act as reflectors at a well-defined wavelength [16].

Most of the sensors mentioned above detect changes in refractive index for small light wave propagation lengths. Their detection limits are therefore not optimal, except for SPR sensors, which are based on an electronic resonance. In optics, a wide range of configurations display optical resonances resulting from a “recycling” of light by multiple passages in a closed space, called a resonator, involving one or more standing waves. Resonances occur at specific wavelength values for which the interference between the transverse waves in the resonator is constructive. If losses in the resonator are low, optical signals can be recycled a large number of times, thus increasing the intensity of the light flowing through the structure at resonant wavelengths. The corresponding number of round trips is related to the quality factor Q of the resonator.

Different types of optical resonances can be exploited in microresonators. In microspheres [17] and microdisks [18], the light is reflected only from the outer walls of the microresonator; these are unguided “whispering gallery modes” (WGM). Recently, Zhang et al. reported the use of micro-bottle cavity biosensors with a high quality factor for DNA detection [19]. Liquid-core optical ring resonators (LCORR) have also demonstrated excellent DNA sensing performances [20]. On the other hand, waveguides with microtores [21] and microrings [22] have also been developed. Photonic crystal structures [23] work as unguided cavities, but they can be coupled with a “photonic guide” consisting of a rectilinear defect in the crystal, located near the cavity. Finally, Fabry–Perot interferometers [24] can be miniaturized, but are difficult to integrate into an optical guide circuit.

Among these various types of optical microresonators, microspheres display very high Q factors. The main difficulty in using them is implementing coupling in order to inject and then recover the resonant light signal in the cavity. These structures are difficult to integrate into optical integrated circuits. For this reason, the most promising candidates for the detection of chemical or biochemical species in integrated optics are microrings and, in a more distant future, photonic crystals.

Table 1 below shows the detection performances of some photonic biosensors for DNA and related molecules (after Reference [25]). We report detection limits for both surface (in pg/mm^2^) and volume (refractive index units or RIU) sensing.

There is still little work on biochemical sensors based on polymer ring microresonators. SU-8 microrings exhibiting a large Q factor (6 × 10^5^) have been developed for the detection of bovine serum albumin (BSA) [22] and showed a detection limit of 12.7 pg/mm^2^, a modest performance as compared to lower Q microrings made by our group using the same SU-8 polymer for TAMRA cadaverine detection (0.05 fg/mm^2^) [33]. This is due to the fact that the results obtained in Reference [22] were obtained with laterally coupled microresonators, contrary to TAMRA cadaverine detection that used vertically coupled microrings.

### 1.2. Detection Schemes in Evanescent Wave Microring Sensors

**Figure 1:**
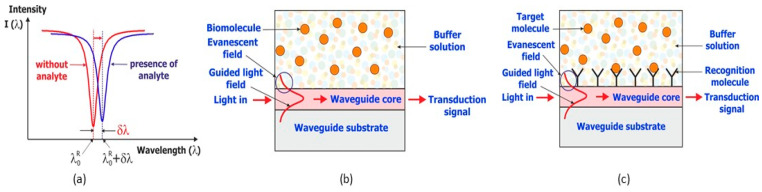
(**a**) Resonant wavelength shift due to the replacement of a pure solvent by a solution of the molecule of interest (analyte). (**b**) Principle of homogenous detection of molecules homogeneously dispersed in a solvent. (**c**) Principle for the surface detection of molecules adsorbed at the core–solution interface.

In waveguide ring-type microresonators, the modification of the effective refractive index of a guided mode is highly sensitive to changes in the composition of the external medium (which is equivalent to cladding in optical fibers). Interactions between the evanescent wave at the core-cladding interface and the moieties contained in this medium induce a modification of the microresonator response in amplitude and in phase. The microresonator resonance wavelengths are then shifted when replacing a reference solvent (usually pure water or a buffer–water solution) with a solution containing the target moiety (analyte) (see Figure 1a). As a consequence, the experimental determination of this wavelength shift provides quantitative information about the concentration of the species of interest in the solution. For this purpose, a calibration must be carried out to determine the dependence of the signal on known concentrations of the compound in solution.

If the molecules to be detected are uniformly distributed in the solution, and if the surface state of the core/solution interface is not modified by the presence of the species to be detected, sensing is performed in a homogeneous detection regime (Figure 1b).

However, this method does not allow for the measurement of very low concentrations, as the spatial extent of the evanescent field, which does not exceed the optical wavelength, remains very small compared to the total volume of the solution. In most cases, commercial index-measuring instruments (the Abbe refractometer, for example) are preferred. Another issue, actually the most important one, is the lack of specificity of the detection process: If the solution to be studied contains several species of varying or unknown concentrations, then their respective contributions to the solution refractive index changes cannot be known.

In view of these limitations, surface detection, during which the analyte molecules to be detected can be adsorbed at the core/cladding interfaces of the waveguide, allows for measurements with a much better detection limit combined with strong selectivity. In other words, the analyte can be detected and quantified in a complex solution where other chemical species are present. For these two reasons, this solution represents the most interesting application of evanescent field sensors (Figure 1c).

However, it is necessary to attach recognition molecules to the surface of the waveguide core, which requires the synthesis of these molecules and grafting chemistry on the surface. This step is absolutely crucial in order to optimize both the specificity and sensitivity of the detection process. These sensors are based on a specific transducer converting a change in chemical structure into a change in optical signal.

### 1.3. Interest of Vertical Coupling in Microring Sensors

**Figure 2:**
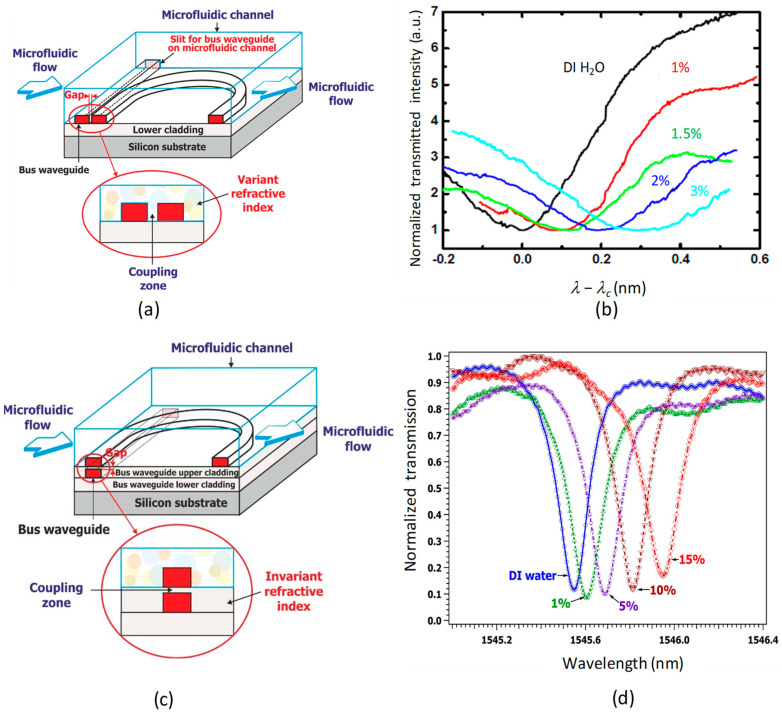
Lateral coupling of a microracetrack with a bus waveguide (**a**); homogeneous glucose sensing with lateral coupling [36] (**b**); vertical coupling of a microracetrack with a bus waveguide (**c**); homogeneous saccharose sensing with vertical coupling [37] (**d**).

There are two types of coupling between the straight guide allowing for light injection into the microring (bus waveguide) and the resonator: lateral coupling and vertical coupling. In lateral coupling, the straight guide and the microring cavity are laterally aligned within the same plane of the lower confinement layer (see Figure 2a). This type of coupling requires a single deposition step only, resulting in coplanar straight and microcavity waveguides showing the same height and material composition. However, this configuration requires strict control of the gap between the straight guide and the microring, with a low roughness in the coupling zone and an excellent parallelism between both guides. A lateral resolution of about ten nanometers is needed, atttainable by electronic beam lithography only.

In vertical coupling, the bus waveguide and the cavity are superimposed in two distinct planes (see Figure 2c), which requires the use of at least two fabrication steps with possibly different materials. A first polymer layer is used to fabricate a buried straight guide corresponding to the bus waveguide, thereby allowing for the injection and extraction of the optical signal into from the resonator. Then, a second layer of another polymer is deposited on the bus guide in order to provide both its upper confinement layer and the lower confinement layer of the microring. The fine control of the gap between two waveguides is ensured by reactive ionic etching (RIE). Finally, a third layer using the same polymer as for the first is deposited on the gap layer to create the optical resonator. The quality of the coupling then strongly depends on the surface condition of the gap layer. Despite a multi-step technological procedure, the vertical coupling configuration has the following advantages: (i) The coupling quality between the buried straight guide and the resonator can be improved as compared to lateral coupling since the vertical gap can be easily controlled by deposition and dry etching. In addition, the coupling quality is less sensitive to a weak depart from parallelism between two vertically aligned surfaces [33,35]. (ii) In lateral coupling, besides high-resolution lithography, the microresonator often needs post-fabrication treatment in order to reduce the surface roughness of the waveguides and gain better control of the gap [32], a process which is not necessary for vertical coupling.

Moreover, in the context of optofluidic sensors, the vertical coupling configuration is particularly advantageous because the coupling region (Figure 2c) is completely isolated from the analyte solution, as the bus waveguide is buried under the gap layer, which is not the case with lateral coupling (Figure 2a). In the latter case, the coupling region contains the solution whose optical index varies according to the analyte concentration. When this concentration changes, the refractive index of this zone is modified, therey perturbing the coupling condition between the two guides. As a result, the quality factor of the microresonator response is poor, with a large degradation of the transducer performance. Figure 2b shows resonance curves of a laterally coupled polymeric microresonator in the homogeneous detection regime for glucose–water solutions whose mass concentrations vary between 0% and 3% [36]. A strong degradation of the quality factor and contrast of the transducer line is observed when increasing the analyte concentration. This situation is not found in the vertically coupled configuration of the microresonator. Indeed, Figure 2d shows the response of a vertically coupled polymer microresonator for the homogeneous detection of a sucrose–water solution with a concentration varying between 0% and 15% by mass [37]. Despite a very high analyte concentration compared to the previous case, the quality factor and contrast of the transducer line change very slightly (up to 10%). A noticeable change is observed only for the highest (15%) concentration. The performance of vertical coupling is much better in this context.

Another important feature for the design of a microring-based optical sensor is a fine control of the coupling between the resonator and the bus waveguide. Choosing a racetrack geometry made of two half-circular waveguides connected to two straight ones provides a coupling zone made of two straight parallel waveguides. This configuration offers an additional degree of freedom for fine control of the coupling coefficient between the bus and the microring, which depends on the length of this coupling area.

In this paper, we report on the fabrication of a micro-racetrack-based optofluidic resonator and its functionalization by a recognition DNA strand for the specific detection of a complementary DNA target oligomer in water. By measuring the device spectral shift for different concentrations of the target DNA, we record a calibration curve obeying the Langmuir model for surface adsorption processes.

## 2. Materials and Methods

### 2.1. Molecules and Materials–Functionalization

The bus waveguide and the micro-racetrack are made of SU-8, a negative photoresist developed by IBM [38]. Due to its good photochemical sensitivity, chemical stability and compatibility with many materials, including biomaterials, SU-8 is widely used in microelectronics. Being transparent in the visible and near-IR range, it can also be used in optical components. Finally, it is compatible with easy surface functionalization after activation by a UV/ozone radiation association. This material consists of a polymer resin initially containing a tetramer of a bis-phenol A derivative functionalized at both ends by an epoxy ring.

The polymer used to form the gap between the bus and the microring is Cytop, a fluoro-copolymer commercialized by AGC Asahi Chemical Corporation. It has been chosen for its high transmittance over a wide spectral range, its ease of deposition in thin films and its small refractive index, close to that of water at 1550 nm (n_cytop_ = 1.335 at 1550 nm). It is used as the top layer for the buried rectilinear waveguide and as the bottom layer for the racetrack microresonator.

The optical transducer is embedded in a microfluidic circuit made of PDMS, a polydimethylsiloxane ([O-Si(CH_3_)_2_]n), i.e., organic silicone polymer. It is viscoelastic, inert, non-toxic, transparent, and displays low swelling in the presence of water or ethanol, which makes it a popular material for the manufacture of microfluidic devices.

The DNA strand attached to the surface of the microresonator as probe DNA is NH_2_-(CH_2_)6-5′-GATACTTCTATCACC-3′. The target complementary DNA, measured in the analyte, is 5’-GGTGATAGAAGTACT-3’. Both DNA strands were synthesized by the Eurogentech company [39]. The objective of the microresonator surface functionalization is to obtain a specific surface detection by the microring immersed in the analyte. In order to facilitate the covalent bonding of the probe DNA to the surface of the microresonator, an amine group was grafted onto this molecule.

**Figure 3:**
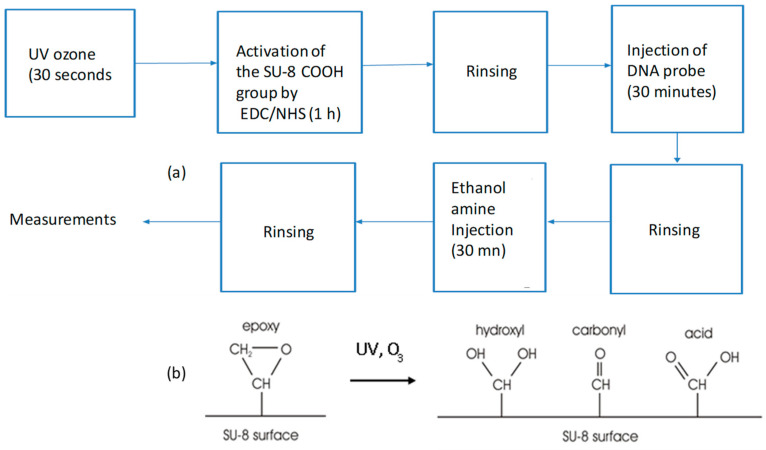
(**a**) Experimental procedure for the functionalization of the microresonator surface. (**b**) UV–ozone oxidization reactions occurring at the SU-8 surface.

The protocol for the functionalization of the SU-8 transducer surface is shown in Figure 3a. First, the transducer is exposed to ozone and UV light in a UV–ozone cleaner for 30 s [40]. This exposure oxidizes the surfaces of the transducer, making them more hydrophilic and facilitating the contact sealing of the microfluidic cap on the optical microresonator when inserted into the microfluidic circuit. On the other hand, it makes the SU-8 surface more reactive by opening the epoxy ring, which induces the appearance of alcohol (OH), aldehyde (CHO) and carboxylic acid (COOH) functions, as shown in Figure 3b.

The binding reaction of the recognition molecule (here the “probe DNA” strand) is based on the amidation of the carboxylic acid functions appearing through the action of UV ozone. These functions need to be made more reactive. For this purpose, an activation process is carried out using EDC (1-Ethyl-3-[3-dimethyl-aminopropyl]carbodiimide hydrochloride), which converts the carboxylic acid function into the more reactive iso-urea, in the presence of a catalyst, NHS (N-hydroxysuccinimide). Then, after rinsing the EDC/NHS solution, the probe DNA solution can be injected at a concentration of 10 µmol·L^−1^. The probe DNA strand was previously modified by adding an amine group to one end of the strand, allowing it to form a covalent bond with the activated carboxylic acid. For the functionalization, we used a solution consisting of 9.7 mg NHS and 42.9 mg EDC dissolved in 1 mL of deionized water. This solution was injected into the microfluidic circuit for one hour. The covalent attachment of DNA on the surface by the formation of the amide link allows high and longterm stability without any loss of chemical properties for further DNA hybridization.

In order to prevent the non-specific detection of the sensor, the areas not occupied by the probe DNA after grafting are filled in with ethanolamine, a very small molecule whose chemical affinity towards the probe DNA and its complement is negligible. The amine group of this molecule is able to form a covalent bond with the activated surface of the transducer as in the case of probe DNA. After rinsing the transducer surface to remove non-grafted probe DNA, we inject a concentrated solution of ethanol amine and leave it in contact with the transducer for sufficient time (30 min) to complete the chemical reaction of its grafting onto the unoccupied surfaces of the transducer. Based on our previous research [41], the alcohol group in the surface of the biolayer gives a negative charge that prevents non-specific interaction. The final step is the rinsing of the transducer surface in order to remove residual ethanol amine molecules from the optofluidic cell.

### 2.2. Microresonator Fabrication

**Figure 4:**
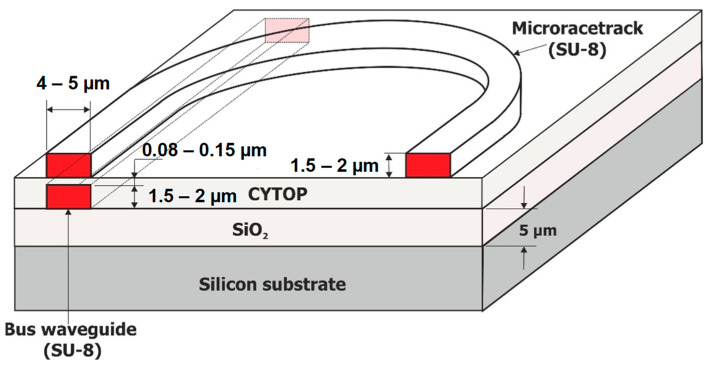
Structure of the polymer-based optical transducer for our optofluidic sensor.

As discussed in the introduction, the polymer optical transducer configuration is a vertically coupled micro-racetrack with the buried bus waveguide on the first level and a microring-shaped microresonator on the second level. The structure of our polymer optical transducer is sketched on Figure 4.

**Figure 5:**
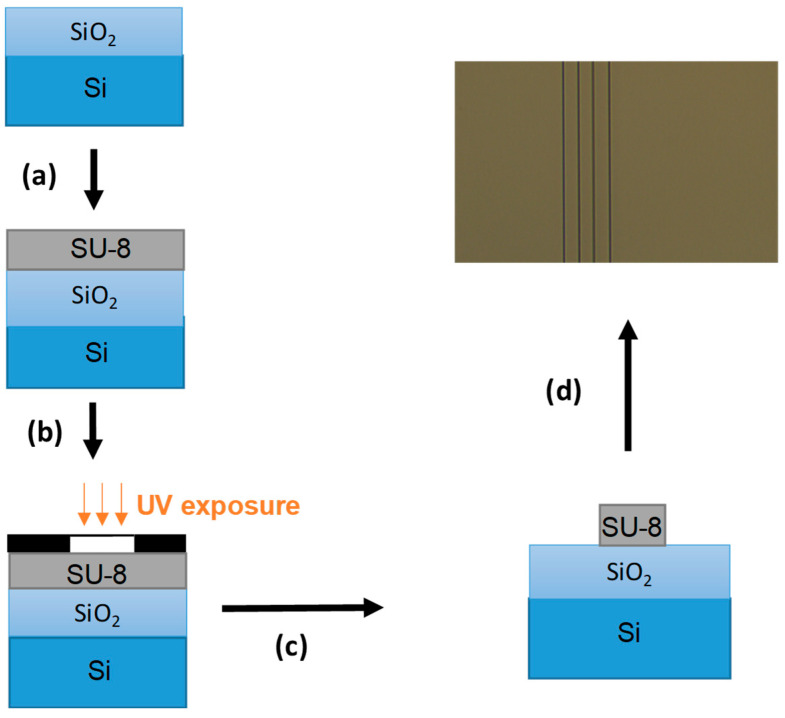
Different steps of SU-8 bus waveguide fabrication: RIE activation and SU-8 deposit by spin coating (**a**); annealing and UV photolithography (**b**); development (**c**); annealing and bus waveguide microscope image (**d**).

The successive steps in the fabrication procedure of the first transducer level are shown in Figure 5. First, the substrate is activated by RIE to facilitate adhesion of the photoresist. Then, the resin is deposited by spin coating (a). After deposition, the sample is annealed on hot plates. It is then exposed to UV light under an optical mask to allow for the creation of the guides of the desired geometry and is annealed again (b). Finally, the sample is developed (c) and annealed (d) in an oven for 2 h in order to stabilize the desired final structure. A photograph of the resulting straight waveguides is taken under an optical microscope.

**Figure 6:**
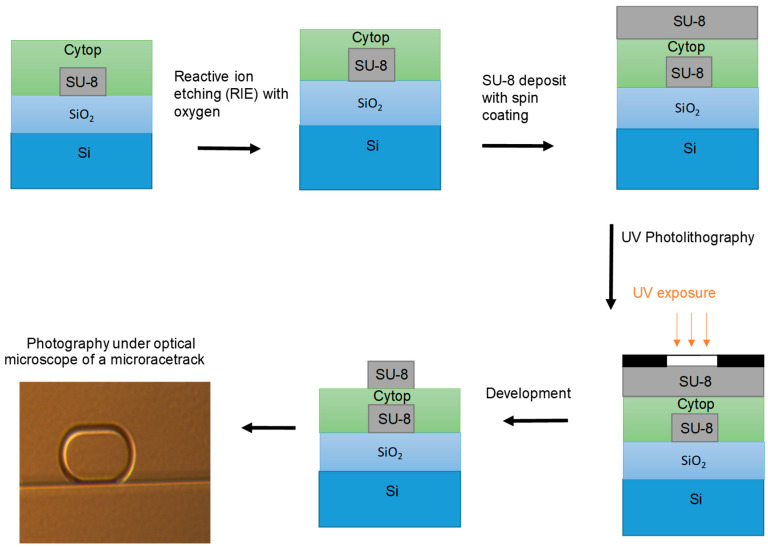
Different steps of SU-8 micro-racetrack fabrication.

The successive steps in the manufacture of the second level of the transducer are shown in Figure 6. First, a layer of Cytop is deposited on the surface by spinning (a). This layer acts as the upper confining layer of the straight waveguide and, at the same time, as the lower confining layer of the micro-racetrack waveguide (Figure 4), so that only the surface of the resonator, in contact with analyte, will be considered as the measurement surface. In addition, the Cytop layer allows for the coupling between the straight waveguide and the resonator to be adjusted by its thickness (gap). Above the Cytop, the micro-racetracks are produced by photolithography, their rectilinear parts of which being aligned with the buried rectilinear waveguides.

### 2.3. Optofluidic Cell: Fabrication and Operation

The microresonators are then inserted into a microfluidic circuit in order to be brought into contact with the fluid to be analyzed. This circuit, a “cap” fixed on the edges of the transducer (i.e., on the Cytop layer), is made of an elastic and inert polymer, polydimethylsiloxane (PDMS), and is elaborated by molding using a SU-8 pattern placed on a silicon wafer. The resulting channel is 26 µm high, 1 mm wide and 12 mm long. The fluid flow in the circuit is controlled by a microfluidic pump and computer-controlled electro-valves.

**Figure 7:**
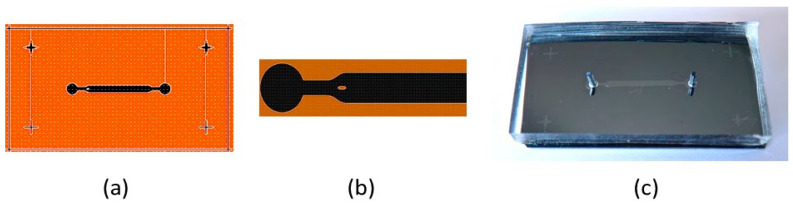
Scheme of the mold used for the fabrication of a microfluidic channel on a Cytop substrate. (**a**) General view; (**b**) detail of the channel entrance showing the elliptical stud; (**c**) photograph of the microfluidic circuit covering the microresonator.

The main challenge of this step is related to the very low wettability of the Cytop fluoropolymer with respect to most solvents, especially water. It is therefore necessary to design a channel shape that avoids the appearance of “dead volumes” in the circulation of the liquid as well as to make the distribution of the velocity fields in the channel as homogeneous as possible. For this purpose, we carried out simulations that allow us to propose and develop a channel whose walls adopt a curvature that follows the flow lines. In addition, an elliptical stud is inserted at the entrance of the channel in order to eliminate dead volumes and to ensure maximum wettability of the liquid on all the walls of the circuit, including on the Cytop surface. Figure 7 shows the shape of the mold used for the elaboration of the microfluidic channel, with an enlarged view of the channel input.

**Figure 8:**
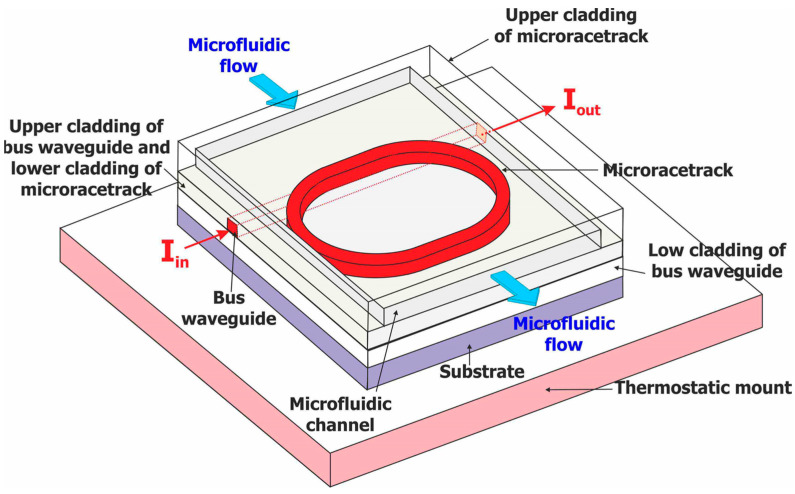
Scheme of our optofluidic cell made of a vertically coupled polymer optical microresonator embedded in a microfluidic channel (from Reference [40]).

The general configuration of our optofluidic cell, as proposed in Reference [39], is shown in Figure 8. It consists of integrating the microfluidic channel in the Cytop polymeric surface. The analyte solution acts as the upper containment layer of the racetrack. The direction of the microfluidic flow is perpendicular to the direction of the optical flow guided by the rectilinear guide.

**Figure 9:**
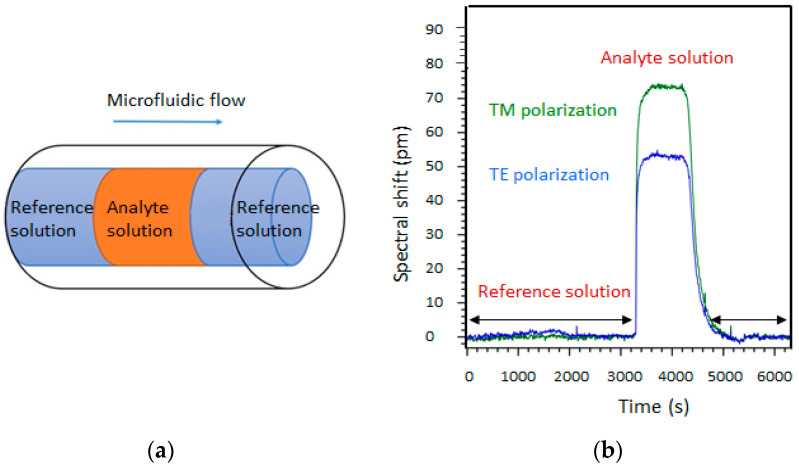
(**a**) Sequence of solution injection in the microfluidic circuit. (**b**) Spectral shift due to the injection of 2.5% glucose by mass into deionised water. Glucose is injected at t = 3200 s.

The routing and control of the microfluidic flow in the optofluidic cell is ensured by a microfluidic station. Reference or analyte solutions are successively introduced into the microfluidic channel of the sensor by a microfluidic pump (Figure 9a). To ensure this continuous flow, one syringe pushes the solution into the sensor, while the other syringe draws it out. A computer-controlled valve is used to send the reference solution into this injection loop in order to push the analyte into the sensor’s microfluidic channel.

The spectral shift of the resonance peak can then be measured after reaching the concentration equilibrium (from 3200 s to 4800 s in Figure 9b), and the concentration of the analyte can be deduced from these data from a sensor calibration curve.

### 2.4. Optical Experimental Set-up

**Figure 10:**
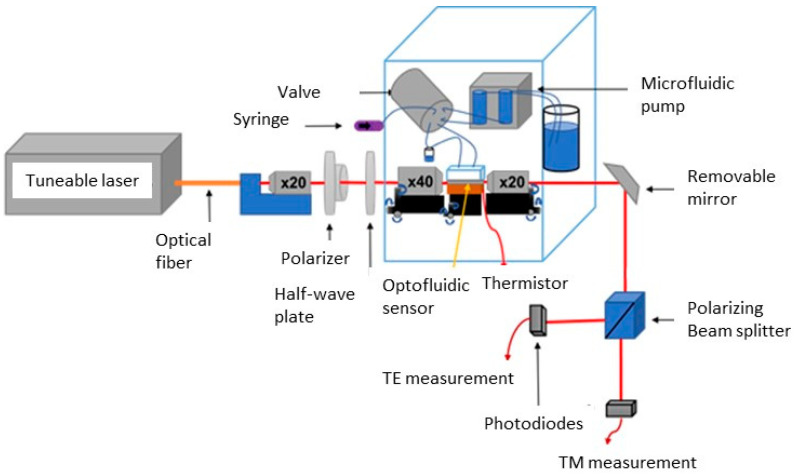
Simplified scheme of the experimental setup. The optical part follows the path of the laser beam (in red); the microfluidic space is contained in the blue cube. The infrared camera is not represented.

The optofluidic measurement bench in the laboratory (Figure 10) is composed of two distinct parts: an optical circuit (shown in red) and a microfluidic part controlling the liquid flow through the optofluidic sensor cell. The optofluidic sensor and the microfluidic station are located in a thermally controlled box.

The optical part is composed of a tunable laser, an optical fiber, a collimation block for the beam exiting the fiber, a polarization block, two microscope objectives for injecting the laser beam at the input of the transducer and for collecting the optical signal at its output, a removable mirror, and a TE and TM polarization splitter and two photodetectors. The Tunics tunable laser (Yenista T100R) allows for wavelength tuning between 1530 and 1630 nm for a 10 mW output power delivery. The typical repeatability of the laser is ±1 pm, and the spectral resolution is ±1 pm.

The output beam of the laser is collected by a single-mode optical fiber with an FC/APC connector. A microscope lens (×20 magnification) is placed at the output of the optical fiber in order to provide a collimated beam. The polarization angle of the incoming beam is selected by a polarizer and a broadband half-wave plate centered at 1550 nm. We chose a polarization of 45° with respect to the vertical axis in order to analyze the output optical signal along TE and TM polarizations. This polarized laser beam is injected into the rectilinear waveguide of the microresonator using a microscope objective of ×40 magnification mounted on an XYZ optical and a piezoelectric stage. The output optical beam is collected by a second microscope objective of ×20 magnification, which directs it to a removable mirror.

In the absence of this mirror, the output optical beam is directed to a near-infrared camera (not represented in the Figure) in order to check that the injection of the input beam into the waveguide has been optimized. When the mirror is present, the optical beam is sent to a polarization splitting cube, allowing for its analysis along TE and TM polarizations. The two corresponding optical signals are directed on two measurement photodiodes (Figure 10).

The tunable laser wavelength, the microfluidic flow, the data acquired from the two photodiodes (TE and TM output signals) and the real-time temperature measurement of the optical transducer are computer-controlled using a homemade software designed and developed in the laboratory [33,40] and programmed in C language provided by the commercial software LabWindows from National Instruments. From this user interface, we can quickly control the parameters of the experiment in real time.

Measurements are performed in a dynamic regime: the reference (or buffer) and the analytesolutions are continuously circulated in the optofluidic cell. This ensures that the pressure remains constant during the measurement process, and that the observed spectral shifts are due solely to the variation in the refractive index of the solution at the liquid-microring interface. The temperature of the optical transducer is measured by a thermistor placed in thermal contact with the transducer, simultaneously with the acquisition of the optical sensor response.

### 2.5. Data Processing

#### 2.5.1. Correction from Temperature Drift

Optical microresonators made of polymeric and inorganic materials are very sensitive to temperature due to their thermo-optical properties and the thermal expansion of the materials. When the temperature of the microring increases, due to thermal contact with its surroundings or the absorption of thermal radiation, a blue spectral shift of the resonance wavelength appears. The variation of this spectral shift is linear in temperature. In the case of homogeneous detection, we have measured the temperature dependence of the sensor spectral response in a static (without liquid flow) mode of deionised water. Thanks to our thermostatically controlled setup, it is possible to stabilize the temperature of the microresonator over a short period of time during which we measure its spectral shift with respect to its response at a reference temperature. The homogeneous thermal drift coefficient *a* of the transducer is defined as *a* = δλ/dT, where δλ is the spectral shift of the optical transducer resonance for a temperature variation dT. We have found that a_TE_ = −86.625 pm/K (resp. a_TM_ = −85.675 pm/K) for TE and TM polarizations, respectively.

During the homogeneous detection of dissolved species in deionised water, we can therefore subtract the homogeneous thermal drift during the measurement. After this correction of the sensor thermal drift, the response returns to the baseline level.

For surface measurements, the optical signal delivered by the sensor is a composite signal containing a homogeneous and a surface component, the latter being related to changes in the local index of the transducer surface. Therefore, the thermal drift of the sensor also shows two contributions. Data processing in this detection mode becomes more complex, as both drift components must be taken into account at the same time. We assume that the surface thermal drift also varies linearly with temperature over a limited temperature variation (a few K) as a first-order perturbation. We can also assume that, in the absence of other disturbances (fluid pressure, mechanical vibration of the sensor, long term drift of the laser source, etc.), the time behavior of the baseline is invariant at a constant temperature. Based on these assumptions, we can process the sensor response data based on the transducer temperature measurement in two steps. The first one consists in subtracting the homogeneous thermal drift as presented above. After this step, the response still contains the surface thermal drift; its baseline does not yet return to zero as desired. In order to correct this drift, we perform a linear regression against the variation of the transducer temperature. This correction is made on limited temperature intervals of the response curve, resulting in a perfectly horizontal baseline illustrating the removal of the thermal drift contribution.

#### 2.5.2. Determination of the Resonance Wavelength Shift

The source emits an optical wave whose wavelength varies linearly with time between two extreme values, *λ_min_* and *λ_max_*. The scanning time τ_scan_ is therefore (*λ_min_ − λ_max_*)/V, where V is the scanning speed. After an initial sweep over a wide spectral range, which allows for the free spectral range of the cavity to be determined, the measurement is restricted to a wavelength range containing only one resonance peak. The spectral shift between the optical response of the reference and analyte solutions can be used to determine the variation of the “measurand”, i.e., the physical quantity *M* (effective refractive index), which is itself linked to the the effective index *n_eff_* and, hence, to the concentration of the analyte, according to the relationship:

$$\delta M=\frac{Kn_{eff,c}}{\lambda_{c}}\delta \lambda$$

where *K* is a constant and *λ_c_* the resonance wavelength.

In general, when using microresonators as detection devices, the method consists in determining the position of an extremum (here, the minimum value of the signal transmitted by the bus waveguide after coupling with the microring) in order to measure the spectral shift due to the variation in liquid composition. If very small quantities of analyte are to be detected, it is essential to optimize the signal-to-noise ratio.

In this work, we have implemented a wavelength shift-pointing methodology involving the slope of the transmission function consisting of determining the wavelength, not at the minimum of the transmission function, but for a value corresponding to the maximum of the slope *p_0_*, i.e., at the inflection point of the response function.

**Figure 11:**
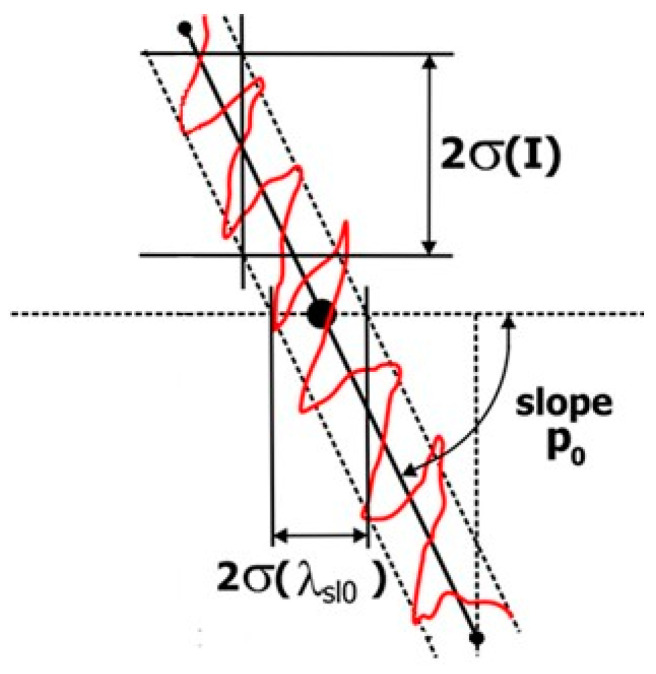
Influence of intensity fluctuations on the standard deviation *σ*(*λ_sl_*_0_) of the wavelength measurement at a point where the transmission function can be considered as linear, with slope *p*_0_.

The signal detected at the output of the microresonator is affected by noise of various origins. A wavelength variation can be considered as significant if, in a continuous measurement, it excesses the standard deviation of the baseline by a factor of 3. This standard deviation depends not only on the wavelength fluctuations *σ(λ)* but also on the intensity noise *σ(I)*, as shown in Figure 11. The uncertainty of *λ* due to *σ(I)* is noted as *σ_int_(λ)*, so the total standard deviation of the wavelength variation measurement can be written as:

$$\sigma \left[\delta \lambda \right]=\sqrt{\sigma^{2}(\lambda )+\sigma_{Int}{}^{2}(\lambda )}$$

On Figure 11 we can see that *σ_int_(λ) = σ(I)/p*_0_, hence:

$$\sigma \left[\delta \lambda \right]\propto \sqrt{\sigma^{2}\left[\lambda \right]+{\left(\frac{\sigma (I)}{p_{0}}\right)}^{2}}$$

As long as the wavelength fluctuations due to the laser source are not dominant, the detection limit can be optimized by working at the inflection point of the transmission function, for which the slope is maximum. This method allows us to obtain a detection limit 2.3 times lower than when pointing the wavelength at the transmission minimum. The steepest possible slope is therefore required. In addition, unlike the method of measuring the spectral shift at the transmission minimum wavelength, a very narrow transmission peak is not required.

Further improvements can be obtained by measuring the spectral shift on both sides of the resonance line and averaging the two measurements. If the line is symmetrical, the noise is reduced by a factor of about $\sqrt{2}$. Finally, as the tunable source operates in a continuous sweep with a return to the initial wavelength after the measurement, it is possible to record the spectral shift, not only during the “forward” phase of the sweep, but also during its “reverse” phase. This procedure does not extend the measurement time but provides more data. As expected, the improvement is close to $\sqrt{2}$. Finally, by measuring at two inflection points and during both the forward and reverse phases of the spectral scan, the resolution of the spectral shift is further improved by a factor of 2.

## 3. Results

### 3.1. Characterization of the Microresonators

**Figure 12:**
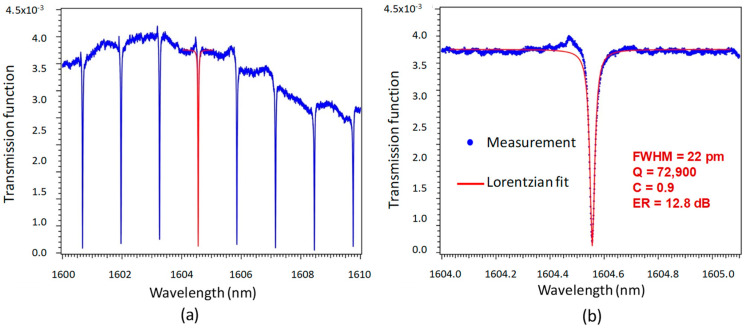
(**a**) General transmission function of a microresonator in air; (**b**) enhanced view of the resonant transmission peak highlighted in red in Figure 12a.

We have tested the microresonators elaborated in our lab in air and in deionised water. Their best characteristics are shown in Figure 12a,b. Figure 12a shows the spectral response of the microresonator over a broad (10 nm) spectral range, while Figure 12b shows the measurement and the fit of the resonance by a Lorentzian fitting function for the resonance peak of Figure 12a highlighted in red. This response is single mode and symmetrical. The wavelength of the resonance peak is 1604.556 nm with a half-value width of FWHM = 22 pm, a quality factor of Q = λ_res_/FWHM up to 72,900 for a contrast of C = 0.9 corresponding to an extinction ratio of ER = 12.8 dB. This performance is the best ever achieved with the optical polymer-based microrings that have been manufactured so far. The straight guide and micro-racetrack widths are 4 µm, the height of the bus waveguide is 1.8 µm, and the racetrack guide height is 1.9 µm. We chose a gap thickness of 77 nm and a racetrack curvature radius of R_c_ = 160 µm. The coupling length between the bus waveguide and the microring is L_c_ = 120 µm.

### 3.2. DNA Functionalization and Recognition on a SU-8 Planar Surface: AFM Studies

In order to characterize the functionalization efficiency of the SU-8 surface after reaction, AFM has been performed on a thin layer of SU-8 (thickness 1.8 µm) deposited on a silicon wafer, as AFM studies are difficult to conduct directly on the surface of the microresonator.

All AFM measurements were realized in tapping mode with a soft tip of 10 nm apex using an Innova AFM from Bruker. Measurements were conducted in three phases. First, we characterized the SU-8 surface deposited on silica. Second, the SU-8 surface was activated by 30 s of UV–ozone treatment, then exposed to the EDC/NHS solution for one hour, and then rinsed with deionised water. This activated SU-8 surface was put in contact with a 2 µmol·L^−1^ solution of probe DNA for half an hour, rinsed and gently dried with dry nitrogen, and characterized by AFM measurements.

The third step consisted in carrying out the linkage between probe and complementary DNA strands. The functionalized surface was exposed to a complementary DNA solution with a concentration of 2 µmol·L^−1^ for half an hour, then the sample was rinsed and dried with nitrogen before being investigated by AFM.

**Figure 13:**
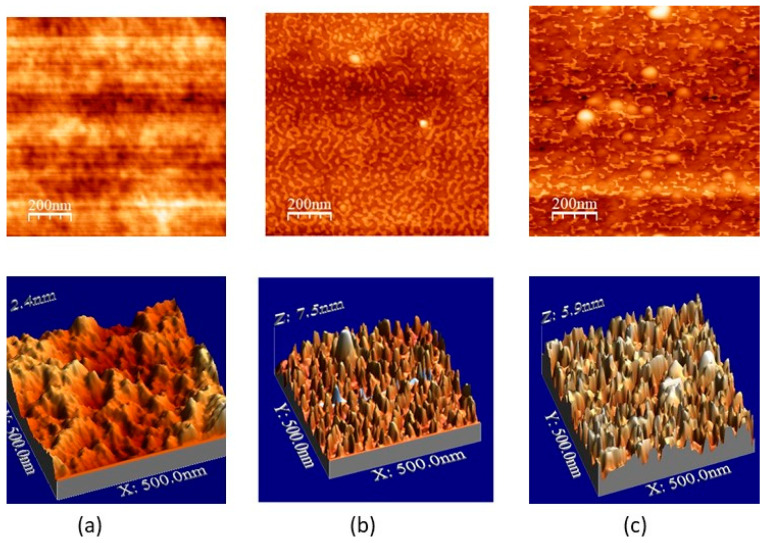
2D (**top**) and 3D (**bottom**) topologies of (**a**) bare SU-8 surface; (**b**) SU-8 surface functionalized by DNA probe; (**c**) SU-8 surface functionalized by DNA probe and its complementary DNA target molecule.

The AFM topologies of the bare SU-8 surface, the SU-8 functionnalized by DNA probe, and the DNA-functionalized SU-8 surface after hybridization with target DNA are shown on Figure 13.

The SU-8 surface before functionalization does not show organized structures in Figure 13a. From the surface profile measurement, the average height variation with respect to the lowest point of the surface does not exceed 0.7 nm, with a 0.12 nm roughness.

In contrast to the case of the bare SU-8 surface, the SU-8 surface functionalized with probe DNA shows structures of a few nanometers visible in Figure 13b. The roughness of the surface is higher that 1 nm, almost 10 times higher than that of the base SU-8 surface. The average height variation is 2.28 nm, which is much greater than that of the bare SU-8 surface, confirming the presence of probe DNA. As the height of the DNA probe used here is about 4.8 nm, the strand’s tilt angle with respect of the normal is 28.3. Finally, after hybridization by the target complementary DNA molecule, the average height is 2.29 nm, and the tilt angle remains almost identical (28.6°).

### 3.3. DNA-Probe Functionalization and Recognition on a SU-8 Planar Surface: Spectral Response of the Sensor

We prepared the probe DNA solution using a phosphate buffer solution (PBS). After activating the SU-8 surface of the microresonator with UV/ozone for 30 s, we injected EDC/NHS into the microfluidic circuit of the sensor so that the activated surface of the microresonator is immersed in this solution for 60 min. Then, the optofluidic cell was connected to the microfluidic station in order to inject the probe DNA solution at a concentration of 10 µmol/L into the microfluidic circuit and a flow rate of 7 µL/min. This functionalization of the microresonator surface with the probe DNA was followed by the injection of the small ethanol amine molecule in order to prevent non-specific detection by the sensor. Finally, the process ended with the injection of the complementary DNA, which is the target molecule to be detected in the sensor.

**Figure 14:**
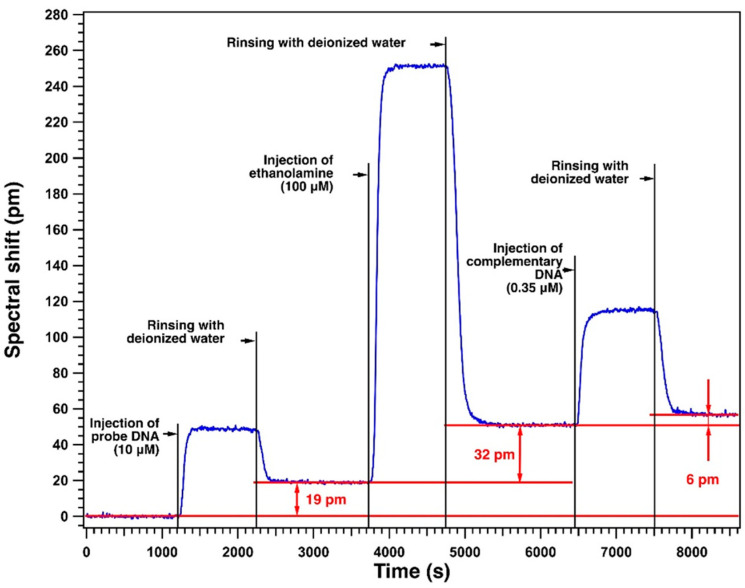
Spectral response of the optofluidic sensor for a complete measurement of DNA hybridization. The first phase is the injection of a 10-micromolar solution of probe DNA onto the surface of the microresonator, already activated by UV/ozone and EDC/NHS. The second phase is the injection of a 100-micromolar solution of ethanol amine. The third phase involves the injection of a 350-nanomolar solution of the complementary DNA.

Figure 14 shows the spectral response of the optofluidic sensor, after correction of the thermal drifts, for a complete characterization of DNA hybridization.

The injection of the probe DNA is detected at 1200 s on the spectral response in Figure 14. The rise time of the spectral response corresponding to the arrival of the probe DNA solution on the surface of the microresonator is quite rapid. At around 1400 s, the composite signal (including the homogeneous and surface detection of this molecule) from the sensor has stabilized. The level of the spectral shift corresponding to this injected concentration of the injected probe DNA is 48 pm relative to baseline. After stabilization of the spectral shift value, rinsing of this surface with deionised water is carried out at 2400 s. The evacuation of the non-functionalized probe molecule is carried out at the same flow rate of 7 µL/min. The duration of this process, dominated by the diffusion of the probe molecule in the buffer rinsing solution, is longer than that of the injection of this molecule on the surface of the microresonator. After 2800 s, the spectral response of the sensor stabilizes at a constant level, reflecting the immobilization of the probe molecule on the surface of the microresonator. The corresponding spectral shift level is 19 pm, (surface detection of the probe molecule). The process of grafting the probe DNA onto the surface of the microresonator is now complete.

The next step aims at preventing non-specific detection using the ethanol amine molecule, which is expected to cover the remaining non-functionalized surface of the microresonator after DNA probe functionalization.

A solution of ethanol amine in deionised water at a concentration of 100 µmol/L is injected at 3720 s. The sensor response increases rapidly, and the spectral shift level stabilizes at around 4000 s with a spectral shift of 231 pm as compared to the level before the injection. After stabilization of the signal, rinsing is performed after 4740 s. The signal corresponding to the surface detection of the sensor stabilizes at 5300 s with a spectral shift of 32 pm, which shows that the small molecule of ethanol amine has filled the surface left uncovered by the DNA probe.

In the final step, a 0.35 µM concentration solution of complementary DNA is injected. For the spectral response of the sensor shown in Figure 14, the injection of this target molecule took place at 6450 s. The composite signal from the sensor is stabilized from 6700 s with a 65 pm spectral shift as compared to the level before injection. After the stabilization of the signal, the sensor is rinsed after 7500 s to remove any target molecules that have not undergone DNA hybridization to the functionalized sensor surface. After rinsing, the spectral shift level stabilized at 6 pm as compared to the level before injection. The DNA hybridization phenomenon takes place and the optofluidic sensor detects the presence of the target molecule at a concentration of 0.35 µM by this specific grafting.

### 3.4. Construction of a Calibration Curve

For a quantitative measurement of an unknown concentration of the DNA target molecule, the relationship between the input value (here the concentration of complementary DNA) and the physical output quantity (here the spectral shift) must be determined in order to evaluate its measurement range, its sensitivity and its detection limit.

**Figure 15:**
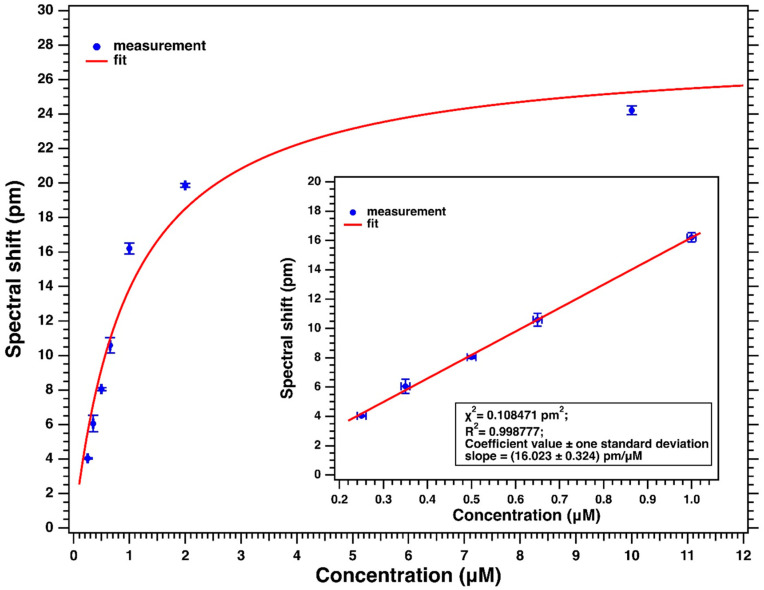
Sensor calibration curve for DNA hybridization measurement. Insert: detail of the linear portion of the calibration curve, providing the sensitivity (S) of our device.

In order to calibrate the optofluidic sensor, we repeated the DNA hybridization measurements as described in Section 3.3. We injected complementary DNA solutions with concentrations ranging from 250 nM to 10 µM at a flow rate of 7 µL/min. For each concentration, we performed at least three different measurements of the corresponding spectral shift on three different microresonators. The calibration curve is displayed in Figure 15. Relative experimental errors do not exceed 10% for spectral shift or 5% for concentration values.

The sensor calibration curve for the detection of DNA hybridization can be fitted using the Langmuir model for surface adsorption reactions according to the following equation

$$\delta \lambda =\frac{\delta \lambda_{max}K_{A}\left[DNA\right]}{K_{A}\left[DNA\right]+1}$$

where *δλ* is the measured spectral shift, *δλ_max_* is the spectral shift obtained for the maximum area covered by the complementary DNA strands (“saturation” of the curve on Figure 15), *K_A_* is the association constant of the DNA hybridization reaction and [DNA] is the concentration of injected complementary DNA. We obtained an association constant of (0.99 ± 0.19)10^6^ mol^−1^/L and a maximum spectral shift of 28 ± 2 pm.

By zooming in on the first few points of the calibration curve, we obtain the response on the linear part of the sensor in the insert of Figure 15. The linear fit of these points provides the sensitivity (S) of the sensor, which is found to reach 16.0 ± 0.3 pm/µM.

We have defined for this sensor a measurement resolution value equal to R = 3σ, where σ is the average of the standard deviations calculated on 100 consecutive measurement points before data processing and on 3 different measurements. We found σ = 0.64 ± 0.1 pm, which leads to a resolution R of 1.94 ± 0.28 pm.

Defining the detection limit as DL = R/S, we obtain a DL value equal to 121 ± 17 nM. As the molecular mass of our target DNA is 4656 g·mol^−1^, the mass concentration detection limit is 563 ± 79 µg/L.

## 4. Discussion

These performances seem to be modest as compared to those obtained using other sensing for the detection of DNA strands, such as quart microbalance, electrochemistry or SPR, as reported in References [6,7,8,9], or FRET-based fluorescence, as reported in Reference [42] for 11 DNA base pairs, for which a detection limit of 50 fM has been evidenced.

However, as the physical origin of surface sensing performed by our microresonators arises from the mass of the material attached on the waveguide surface, a systematic comparison of the limits of detection for different types of DNA sensing is not relevant. The attachment of a heavy molecule containing only one DNA chain is expected to induce a sensing signal that would be of a similar order of magnitude as that due to the same mass of a large number of DNA strands. It could be more adequate to compare detection limit concentrations expressed in mass per surface unit rather than in molar concentrations, as shown in Table 1. However, because our DNA targets do not display any measurable physical properties like DNA labelled with fluorescent probes, we are not currently able, contrary to our previous report on TAMRA-Cadaverine [33,37], to evaluate the mass per resonator surface unit of our hybridized DNA target molecule.

The main advantage of using optofluidic microresonators as sensors is their ability to provide real-time responses, contrary to other techniques. There are very few reports on DNA detection using optical microresonators. In Reference 19, a hollow-core micro-bottle whispering gallery mode-based microcavity sensor showing a high quality factor Q (>10^6^) displays very low detection limits for 10-mer and 25-mer DNA strands (28 pM and 0.64 pM, respectively). However, in their experimental procedure, the authors did not mention any rinsing step after injecting target DNA solutions into cavities functionalized by probe DNA chains. Given that the target DNA solution is not washed-out, the observed signal contains both homogeneous and surface contributions. Contrarily, the LCORR-based DNA sensor reported in Reference [20], with a Q factor of 10^6^, displays a detection limit of 10 pM (or 4 pg/mm^2^) after rinsing. In these two examples, these remarkable performances are related to the high Q values frequently met in WGM resonators. However, even if LCORR and analogous resonators are easy to fabricate and compatible with microfluidic circuitry, their integration into multiplexed platforms compatible with silicon processing technology remains problematic. This is the reason why LCORR have not been widely developed after the first results reported in Reference [20]. In our work, we have been able to integrate both optical and microfluidic circuits on a silicon wafer. This is, to the best of our knowledge, the first demonstration of label-free DNA hybridization sensing using a polymer-based, vertically coupled waveguided microresonator embedded in a microfluidic circuit.

## 5. Conclusions

We functionalized the surface of the microresonators using amine-modified DNA strands. The efficiency of this functionalization is confirmed by the optical response of the sensor as well as by AFM measurements. We were able to hybridize this DNA strand with a complementary DNA strand, and we have inferred the dissociation constant value of the binding equilibrium of the target DNA to the probe DNA. The stability constant of this reaction is 10^6^, which confirms the possibility of achieving DNA strand recognition by the recognition molecules. These results open attractive perspectives for the use of polymer-based microresonators as real-time detectors of DNA hybridization. This work serves as a proof of concept of the ability of the microresonator integrated into a microfluidic cell to follow the kinetics of DNA hybridization. Detection in human bodily fluid is not performed in this study, and future research will be focused on real application of the DNA detection.

It would be worthwhile to implement the following developments and improvements in the future. First, it would be interesting to use longer DNA strands for AFM measurements, specifically to check whether the spectral shift can become higher for greater thicknesses of the hybridized DNA layer on the surface.

On the other hand, measurements involving biomolecules derived directly from living organisms can be envisaged for the future, such as the measurement of microRNAs involved in the development of cancers.

In addition, the experimental setup could also be improved; indeed, we have noted a strong influence of temperature and pressure variations on the spectral shift values. In order to avoid these effects, a differential setup using two parallel microresonators could be used. This setup would provide a reference response of the pure solvent, without analyte, to be compared in real time with the simultaneous measurement of the analyte response. The “reference” signal obtained would allow for the correction of the effects of disturbances that are not related to the presence of analyte on the surface of the microresonator. Therefore, we expect important improvements of the performances of this optofluidic device using this new architecture.

A multiplexing measurement device could also be developed in which several resonators are functionalized with different ligands allowing for the simultaneous detection of several chemical or biological species present in solution. This type of measurement requires the same analyte solution to be fed to all of the microring systems while retaining a reference channel to measure the pure solvent signal. Such a multiplexed device would be very promising for the parallel analysis of different species in solution.

## Figures and Tables

**Table 1:** Comparison of the performances of typical photonic sensors.

Sensor Type	Q	Surface Detection Limit (pg/mm^2^)	RIU Detection Limit	Material	Target Molecule	Ref.
Mach-Zehnder	N.A.	0.06	1 × 10^−7^	Si_3_N_4_	DNA	[26]
Young interferometer	N.A.	0.013	9 × 10^−9^	Ta_2_O_5_	IgG	[27]
Slit waveguide	N.A.	0.05	2.5 × 10^−7^	Si_3_N_4_	BSA	[28]
Microsphere	5 × 10^6^	6	3 × 10^−7^	Glass	DNA	[29]
LCORR	10^6^	4.0	8.8 × 10^−6^	Silica	DNA	[20]
Microring	6 × 10^5^	1.6	2.8 × 10^−7^	SOI	DNA	[30]
Photonic crystal	N.A	0.42	3.4 × 10^−5^	Si_3_N_4_	Antibiotin IgG	[31]
Microring	2 × 10^4^	250	~10^−7^	Polystyrene	Streptavidin	[32]
Microring	3.5 × 10^4^	5 × 10^−5^	5 × 10^−5^	SU-8	5-TAMRA Cadaverin	[33]
Microring	6 × 10^5^	12.7	~10^−5^	SU-8	BSA (Bovine Serum albumin)	[34]

## Data Availability

The data presented in this study are available on request from the corresponding author.

## References

[1] Pelt-Verkuil E., Belkum A., Hays J.P. (2008). Principles and Technical Aspects of PCR Amplification.

[2] Li Z., Yi Y., Luo X., Xiong N., Liu Y., Li S., Sun R., Wang Y., Hu B., Chen W. (2020). Development and clinical application of a rapid IgM-IgG combined antibody test for SARS-CoV-2 infection diagnosis. J. Med. Virol..

[3] Singh A., Sharma A., Ahmed A., Sundramoorthy A.K., Furukawa H., Arya S., Khosla A. (2021). Recent Advances in Electrochemical Biosensors: Applications, Challenges, and Future Scope. Biosensors.

[4] Pieralli C., Attoui C., Boireau W., Wacogne B. (2009). Low cost optical detection of DNA hybridization on biochip. Laser Phys..

[5] Bujalowski W.M. (2012). Spectroscopic Methods of Analysis: Methods and Protocols, Methods in Molecular Biology.

[6] Okahata Y., Matsunobu Y., Ijiro K., Mukae M., Murakami A., Makino K. (1992). Hybridization of nucleic acids immobilized on a quartz crystal microbalance. J. Am. Chem. Soc..

[7] Chen Y., Zhong Y., Ye J.-X., Lei Y., Liu A.-L. (2022). Facile Label-Free Electrochemical DNA Biosensor for Detection of Osteosarcoma-Related Survivin Gene. Biosensors.

[8] Wang J. (1999). Towards Genoelectronics: Electrochemical Biosensing of DNA Hybridization. Chem. Eur. J..

[9] Wang R., Tombelli S., Minunni M., Spiriti M.M., Mascini M. (2004). Immobilization of DNA probes for the development of SPR-based sensing. Biosens. Bioelectron..

[10] Shrivastav A.M., Cvelbar U., Abdulhalim I.A. (2021). A comprehensive review on plasmonic-based biosensors used in viral diagnostics. Commun. Biol..

[11] Nair R.V., Vijaya R. (2010). Photonic crystal sensors: An overview. Prog. Quantum Electron..

[12] Li L., Zhang Y.-N., Zhou Y., Zheng W., Sun Y., Ma G., Zhao Y. (2021). Optical Fiber Optofluidic Bio-Chemical Sensors: A Review. Laser Photonics Rev..

[13] Berneschi S., Nunzi Conti G., Pelli S., Soria S. (2009). Microresonators for Sensing Applications. Series in Optics and Photonics: An Introduction to Optoelectronic Sensors.

[14] Subramanian S., Wu H.-Y., Constant T., Xavier J., Vollmer F. (2018). Label-Free Optical Single-Molecule Micro- and Nanosensors. Adv. Mater..

[15] Chandrasekar R., Lapin Z.J., Nichols A.S., Braun R.M. (2019). Photonic integrated circuits for Department of Defense-relevant chemical and biological sensing applications: State-of-the-art and future outlooks. Opt. Eng..

[16] Chan C.-F., Chen C., Jafari A., Laronche A., Thomson D.J., Albert J. (2007). Optical fiber refractometer using narrowband cladding-mode resonance shifts. Appl. Opt..

[17] Vollmer F., Arnold S. (2008). Whispering-gallery-mode biosensing: Label-free detection down to single molecules. Nat. Meth..

[18] Schweinsberg A., Hocdé S., Lepeshkin N.N., Boyd R.W. (2007). An environmental sensor based on an integrated optical whispering gallery mode disk resonator. Sens. Actuator B-Chem..

[19] Zhang S., Wan H., Xiong J., Wan C., Lu Y., Ang L., Lv P. (2022). A high hollow-core micro-bottle cavity biosensors for DNA detection with low detection limit. J. Light. Technol..

[20] Suter J.D., White I.M., Zhu H., Shi H., Caldwell C.W., Fan X. (2008). Label-free quantitative DNA detection using the liquid core optical ring resonator. Biosens. Bioelectron..

[21] Hunt H.K., Soteropulos C., Armani A.M. (2010). Bioconjugation strategies for microtoroidal optical resonators. Sensors.

[22] Iqbal M., Gleeson M.A., Spaugh B., Tybor F., Gunn W.G., Hochberg M., Baehr-Jones T., Bailey R.C., Gunn L.C. (2010). Label-free biosensor arrays based on silicon ring resonators and high-speed optical scanning instrumentation. IEEE J. Sel. Top. Quantum Electron..

[23] Chakravarty S., Zou Y., Lai W.C., Chen R.T. (2012). Slow light engineering for high Q high sensitivity photonic crystal microcavity biosensors in silicon. Biosens. Bioelectron..

[24] Bergstein D.A., Ozkumur E., Wu A.C., Yalçin A., Colson J.R., Needham J.W., Irani R.J., Gershon J.M., Goldberg B.B., Delisi C. (2008). Resonant cavity imaging: A means toward high-throughput label-free protein detection. IEEE J. Sel. Top. Quantum Electron..

[25] Fernández Gavela A., Grajales García D., Ramirez J.C., Lechuga L.M. (2016). Last Advances in Silicon-Based Optical Biosensors. Sensors.

[26] Zinoviev K., Carrascosa L.G., Sánchez del Río J., Sepúlveda B., Domínguez C., Lechuga L.M. (2008). Silicon photonic biosensors for lab-on-a-chip applications. Adv. Opt. Technol..

[27] Schmitt K., Schirmer B., Hoffmann C., Brandenburg A., Meyrueis P. (2007). Interferometric biosensor based on planar optical waveguide sensor chips for label-free detection of surface bound bioreactions. Biosens. Bioelectron..

[28] Zinoviev K.E., González-Guerrero Domínguez C., Lechuga L.M. (2011). Integrated bimodal waveguide interferometric biosensor for label-free analysis. J. Light. Technol..

[29] Hanumegowda N.M., Stica C.J., Patel B.C., White I., Fan X. (2005). Refractometric sensors based on microsphere resonators. Appl. Phys. Lett..

[30] Li H., Fan X. (2010). Characterization of sensing capability of optofluidic ring resonator biosensors. Appl. Phys. Lett..

[31] Cunningham B., Li P., Lin B., Pepper J. (2002). Colorimetric resonant reflection as a direct biochemical assay technique. Sens. Actuators B Chem..

[32] Chao C.-Y., Fung W., Guo L.J. (2006). Polymer microring resonators for biochemical sensing applications. IEEE J. Sel. Top. Quantum Electron..

[33] Delezoide C., Salsac M., Lautru J., Leh H., Nogues C., Zyss J., Buckle M., Ledoux-Rak I., Nguyen C.T. (2012). Vertically coupled polymer micro-racetrack resonators for label-free biochemical sensors. IEEE Photon. Technol. Lett..

[34] Tu X., Chen S.-L., Song C., Huang T., Guo L.J. (2019). Ultrahigh Q Polymer Microring Resonators for Biosensing Applications. IEEE Phot. J..

[35] Little B.E., Chu S.T., Pan W., Ripin D., Kaneko T., Kokubun Y., Ippen E. (1999). Vertically coupled glass microring resonator channel dropping filters. IEEE Photonics Technol. Lett..

[36] Chao C.-Y. (2005). Polymer Microring Resonators and Its Applications as Biosensors. Ph.D. Thesis.

[37] Delezoide C., Lautru J., Zyss J., Ledoux-Rak I., Nguyen C.T. (2012). Vertically coupled polymer microresonators for optofluidic label-free biosensors. Proceedings of the Integrated Optics: Devices, Materials and Technologies XVI.

[38] Shaw J.M., Gelorme J.D., LaBIanca N.C., Conley W.E., Holmes S.J. (1997). Negative photoresists for optical lithography. IBM J. Res. Develop..

[39] McKean D.R., Schaedeli U.P., Kasai P.H., MacDonald S.A. (1991). The Effect of Polymer Structure on the Efficiency of Acid Generation from Triarylsulfonium Salts. J. Polym. Sci. Part A Polym. Chem..

[40] Chauvin D., Bell J., Leray I., Ledoux-Rak I., Nguyen C.T. (2019). Label-free optofluidic sensor based on polymeric microresonator for the detection of cadmium in tap water. Sens. Actuators B Chem..

[41] Ahmad L., Salmon L., Korri-Youssoufi H. (2019). Electrochemical detection of the human cancer biomarker ‘autocrine motility factor-phosphoglucose isomerase’ based on a biosensor formed with a monosaccharidic inhibitor. Sens. Actuators B Chem..

[42] Megalathan A., Wijesinghe K.M., Dhakal S. (2021). Single-molecule FRET-Based Dynamic DNA Sensor. ACS Sens..

